# Can the Lateral Habenula Crack the Serotonin Code?

**DOI:** 10.3389/fnsyn.2016.00034

**Published:** 2016-10-24

**Authors:** Anna Tchenio, Kristina Valentinova, Manuel Mameli

**Affiliations:** ^1^Institut du Fer à MoulinParis, France; ^2^Institut National de la Santé et de la Recherche Médicale (INSERM), UMR-S 839Paris, France; ^3^Université Pierre et Marie CurieParis, France

**Keywords:** 5-HT, lateral habenula (LHb), synapses, raphe nuclei, neuromodulation

## Abstract

The lateral habenula (LHb) and the serotonergic system both contribute to motivational states by encoding rewarding and aversive signals. Converging evidence suggests that perturbation of these systems is critical for the pathophysiology of mood disorders. Anatomical and functional studies indicate that the serotonergic system and the LHb are interconnected in a forward-feedback loop. However, how serotonin release modifies the synaptic and cellular properties of LHb neurons and whether this has any behavioral repercussions remain poorly investigated. In this review article, we discuss insights gained from rodents and humans regarding the implications of the serotonin system and the LHb in aversion encoding and related disorders. We then describe the type, properties and pharmacology of serotonergic receptors expressed throughout the LHb. Finally, we discuss physiological data reporting how serotonergic signaling modifies synaptic transmission and neuronal activity within the LHb. Altogether, we combine a mechanistic- and circuit-level knowledge to provide an overview on how the LHb integrates serotonergic signals, a process potentially contributing to LHb-dependent encoding of valenced external stimuli.

## Introduction

The lateral habenula (LHb) bidirectionally connects with neuromodulatory centers, including the serotonergic raphe and dopaminergic midbrain nuclei (Wang and Aghajanian, 1977; Christoph et al., 1986; Varga et al., 2003; Hikosaka, 2010). Functional evidence from non-human primates indicates that LHb neurons encode external aversive and rewarding experiences (Matsumoto and Hikosaka, 2007). Furthermore, experimental results from humans and rodents suggest that LHb neuronal hyperactivity may represent a cellular substrate underlying depressive-like symptoms in mood disorders and drug addiction (Sartorius et al., 2010; Shabel et al., 2014; Meye et al., 2015, 2016; Lecca et al., 2016). Altogether, this indicates that the LHb represents a major anatomical substrate, processing valenced stimuli in physiological and pathological conditions.

The serotonergic system also participates in the processing of motivation (Faulkner and Deakin, 2014). A wealth of literature supports the idea that its perturbation contributes to drug addiction (Müller and Homberg, 2015) and, importantly, to the etiology of mood disorders (Cannon et al., 2007). However, whether serotonin-dependent modulation of LHb shapes motivational states remains elusive. Here, we will first describe the anatomical and functional relevance of serotonergic signaling within the LHb. Next, we will discuss the potential significance of serotonin control on LHb function for behaviors associated with motivation and related disorders.

## LHb Processing of Reward and Aversion

The LHb is located in the epithalamus, underneath the third ventricle and above the posterior end of the thalamus. It includes a medial and a lateral division, which are anatomically distinct, receive specific innervation and can encode aversive behaviors (Andres et al., 1999; Shabel et al., 2012; Stamatakis et al., 2016). Indeed, anterograde and optogenetic studies demonstrated that afferents from the entopeduncular nucleus (EPN), the ventral tegmental area or the lateral hypothalamus innervate different territories, and drive aversive behaviors (Shabel et al., 2012; Root et al., 2014; Stamatakis et al., 2016). Neurons located in the LHb are predominantly of glutamatergic type and although morphologically diverse, they are present as simple dendritic arborization and postsynaptic spines (Weiss and Veh, 2011; Maroteaux and Mameli, 2012). Despite this general homogeneous phenotype, LHb neurons present a heterogeneous expression of neuropeptides and proteins, indicating that distinct habenular neuronal subpopulations may serve different biological functions (Geisler et al., 2003; Weiss and Veh, 2011; Aizawa et al., 2012; Proulx et al., 2014). However, the significance of each LHb neuronal subpopulation remains obscure. If a cytochemical identification of LHb neurons exists, the development of genetic tools can allow to discriminate and test the significance of each individual LHb neuronal subtype allowing to refine our knowledge on LHb function.

Glutamatergic projections from the LHb descend through the fasciculus retroflexus and make synapses with neuronal populations in deep structures: GABAergic and dopaminergic neurons in the midbrain and GABAergic and serotonergic neurons in the dorsal and median raphe (DRN and MRN respectively; Wang and Aghajanian, 1977; Stern et al., 1979; Lammel et al., 2012; Stamatakis and Stuber, 2012; Pollak Dorocic et al., 2014).

Seminal studies in behaving monkeys initially described that neuronal activity in the LHb contributes to the processing of aversive and rewarding stimuli (Matsumoto and Hikosaka, 2007). Indeed, the activity of LHb neurons increases when an airpuff (aversive stimulus) is presented in an unexpected fashion. After a series of conditioning sessions, the firing of LHb neurons increases following a cue predicting the aversive stimulus onset. Conversely, the unexpected delivery of rewards, and cues predicting them decreases LHb neuronal firing (Matsumoto and Hikosaka, 2007, 2009). This role of the LHb in processing negative stimuli and “anti-reward” signals is further supported by studies employing optogenetic approaches. LHb infusion with viral vectors encoding for excitatory opsins allows to probe the behavioral importance of LHb output onto afferent midbrain nuclei. Optical stimulation of LHb terminals leads to avoidance behaviors (Lammel et al., 2012; Stamatakis and Stuber, 2012). These findings support the idea that activation of the LHb is sufficient for driving negative motivational states.

However, whether neuromodulators gate this LHb-mediated behavior remains poorly studied. In this review article, we will focus on the role of serotonin-dependent neuromodulation within the LHb.

## Bidirectional Connectivity Between Raphe and LHb

Neuroanatomical tracing studies reported a direct projection from LHb neurons to both DRN and MRN, as well as an indirect projection via the GABAergic rostromedial tegmental nucleus located in the midbrain (Wang and Aghajanian, 1977; Jhou et al., 2009; Bernard and Veh, 2012; Quina et al., 2015). LHb neurons sending axons to the raphe are mainly located in the medial territory of the LHb. Moreover, axons from the LHb more prominently target the MRN compared to the DRN (Bernard and Veh, 2012; Quina et al., 2015). However, the above-mentioned tracing studies did not differentiate the targeted neuronal subtype within the raphe. Recent studies employed rabies-based viral strategy and Cre-driver mouse lines to identify that LHb neurons make synapses onto DRN/MRN serotonergic neurons and DRN GABA neurons (Pollak Dorocic et al., 2014; Weissbourd et al., 2014).

Importantly, also raphe neurons send axons to the LHb. Tracing approaches using phalloidin anterograde labeling indicate the presence of MRN and DRN fibers throughout the whole LHb (Vertes, 1991; Vertes et al., 1999). One of the limitations of this analysis is the lack of information regarding cell-type specificity of the raphe neurons projecting onto the LHb. Using transgenic mice expressing Cre-recombinase in Sert-positive raphe neurons provided evidence that serotonin cells in the DRN send a prominent axonal innervation to the lateral portion of the LHb (Morin and Meyer-Bernstein, 1999; Muzerelle et al., 2016), in line with the presynaptic expression of serotonin and its transporter in the LHb (Kiyasova et al., 2011). This is in stark contrast with other immunolabeling studies indicating the terminal expression of Sert or serotonin itself in the medial portion of the LHb (Geisler et al., 2003; Zhang et al., 2016). The combinatorial use of genetic tools, optogenetic strategies and electrophysiology will be necessary to refine our understanding of the LHb-Raphe-LHb connectivity.

## Serotonin Receptor Expression within the LHb

Serotonin can activate 14 subtypes of receptors, which belong to seven families according to their pharmacological and molecular properties (Barnes and Sharp, 1999). The signaling pathways of the different isoforms have been extensively described (Hoyer et al., 1994). In summary, with the exception of 5-HT3 which is a ligand-gated cation channel, all the 5-HT receptors are G-coupled proteins (Millan et al., 2008). 5-HT1 (5-HT1A-F) and 5-HT5A receptors are predominantly coupled to G_i/o_ proteins, which can inhibit cyclic AMP, open K^+^ or close Ca^2+^ channels. 5-HT2 receptors (5-HT2A-C) couple to G_q_ proteins and increase the Inositol-3-phosphate hydrolysis leading to diacylglycerol generation. 5-HT4, 5-HT6 and 5-HT7, instead, are G_s_-coupled proteins and act to increase cyclic AMP levels. 5HT receptors, depending on the subtype and anatomical localization, can be pre- or postsynaptically expressed, thereby regulating presynaptic neurotransmitter release or postsynaptic cell function, respectively (Barnes and Sharp, 1999).

Several subtypes of 5-HT receptors are expressed in the LHb including the 5-HT1B, 5-HT2C, 5-HT7 and 5-HT5. *In situ* hybridization techniques revealed high labeling of 5-HT7 in the LHb at postnatal day 5 in the rat. However, this labeling decreased over time reaching a very low signal in the adult stage (Vizuete et al., 1997), indicative of a developmental regulation of this receptor subtype. While the 5-HT2A was not detected, a strong expression of 5-HT2C was found throughout the whole LHb (Pompeiano et al., 1994; Clemett et al., 2000). Accordingly, micro-array analysis also revealed 5-HT2C and 5-HT1B expression, the latter mainly localized in the medial part of the LHb (Wagner et al., 2016). Although 5HT receptor expression within the LHb has been widely assessed, their localization at the pre- or postsynaptic compartment, or their functional and behavioral significance remains less clear. Only recently, data obtained using electrophysiological approaches indicated that while the 5-HT2C is postsynaptically expressed, the 5-HT1B is rather presynaptically located, providing the first insight on the physiological role of serotonin within the LHb (Hwang and Chung, 2014; Zuo et al., 2016).

## Serotonin-Driven Modulation of Synaptic Transmission in the LHb

Serotonin release modulates glutamatergic and GABAergic synaptic transmission throughout the central nervous system by acting on its specific receptors (Maejima et al., 2013). Recent evidence indicates that serotonin modulates excitatory and inhibitory synaptic currents onto LHb neurons. An initial set of experiments made use of the expression of Channelrhodopsin-2 in the EPN of the basal ganglia. EPN neurons send axons capable to co-release glutamate and GABA to the lateral portion of the LHb (Shabel et al., 2012). Light-evoked excitatory and inhibitory postsynaptic currents (EPSCs and IPSCs, respectively) were reduced by continuous bath application of exogenous serotonin. The diminished amplitude of EPSCs and IPSCs occurred along with an increased paired pulse ratio, indicative of a presynaptic reduction in the probability of neurotransmitter release (Shabel et al., 2012, 2014). However the subtype of 5-HT receptor involved in this modulation, as well as its pre- or postsynaptic localization remains still unknown.

**Figure 1:**
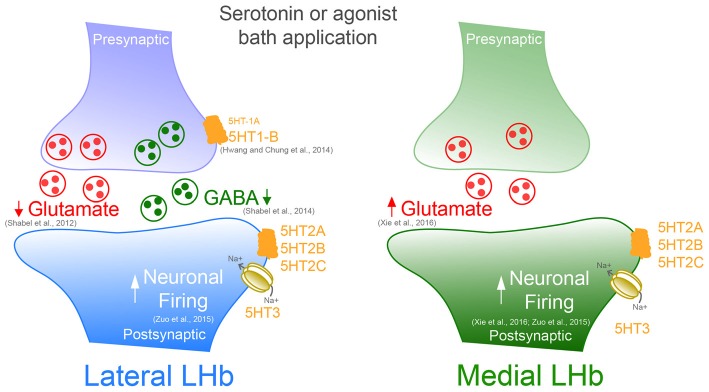
**Serotonin or specific bath application of 5-HT agonists modifies presynaptic and postsynaptic neurotransmission in both the medial and lateral portion of the Lateral habenula (LHb)**.

5-HT1B receptors are expressed in the LHb, are typically presynaptically located and most often control presynaptic neurotransmitter release (Lesch and Waider, 2012; Wagner et al., 2016). The activation of this receptor subtype may therefore underlie serotonin-dependent modulation of EPN-to-LHb synapses. Accordingly, acute exposure of LHb-containing slices to the specific 5-HT1B receptor agonist CP93129 or to serotonin produced: (i) a long-lasting depression of evoked EPSCs; and (ii) a transient reduction of spontaneous EPSC frequency (Hwang and Chung, 2014; Xie et al., 2016). This effect was prevented by the 5-HT1B receptor antagonist SB216641. Notably, an agonist of the 5-HT1A receptors only transiently decreased EPSCs (Hwang and Chung, 2014). However, also this effect was blocked by the 5-HT1B antagonist, indicating either a cross-talk between receptor subtypes or alternatively a non-specific effect (i.e., concentration dependent) of these compounds (Table 1). This form of plasticity was presynaptically expressed, and required nitric oxide and ryanodine receptor-dependent Ca^2+^ release. Importantly, no modifications were observed postsynaptically, as serotonin failed to change AMPA receptor-mediated responses triggered by direct AMPA application (Hwang and Chung, 2014). Whether the reported serotonin-dependent reduction at EPN-to-LHb synapse requires similar mechanisms remains to be established. This work highlights important issues regarding the pharmacology of 5HT, suggesting that the results obtained to date using synthetic compounds should be carefully interpreted given the complex pharmacology of 5HT receptors (Table 1 and Figure 1). Conditional deletion or the use of CRISPR-Cas9 technology to downregulate specific 5HT receptors may represent an alternative approach to provide more precise and informative insights on the type of receptors governing LHb synaptic function (Shalem et al., 2015).

**Table 1:** **Pharmacological effects of 5-HT receptor agonists and antagonists on the synaptic properties of Lateral habenula (LHb) neurons**.

Receptor	Agonist	Effect in LHb	Antagonist	Effect in LHb	References
5-HT1	5-CT	Reduction of EPSCs	Cyanopindolol	Blocks EPSC reduction	Hwang and Chung (2014)
5-HT1B	CP93129	Reduction of EPSCs	SB216641	Blocks EPSC reduction	Hwang and Chung (2014)
5-HT2A-C	α-methyl 5HT	High dose: ↓ EPSCs; low dose: ↑ EPSCs	Ritanserin	Blocks the ↑ of EPSCs induced by a *low dose* of α-methyl 5HT	Hwang and Chung (2014)
	mCPP	Slow inward current; ↑ frequency of sEPSCs ↑ firing rate	Ritanserin SB200646	Attenuates effects of 5HT	Xie et al. (2016) and Zuo et al. (2016)
5-HT3	mCPBG	Slow inward current; ↑ firing rate	OND	Attenuates effects of 5HT	Xie et al. (2016) and Zuo et al. (2016)

In most cells, however, spontaneous glutamate mediated synaptic activity onto LHb neurons was facilitated by serotonin. This effect occurred via increased presynaptic glutamate release through 5-HT2 and 5-HT3 receptor activation (Xie et al., 2016). However, insights about the input-specific expression and subcellular localization of these receptors are still lacking and require further investigation. Future studies would need to provide or rule out whether LHb neurons located in the medial or lateral territory express different 5-HT receptor subtypes. These findings would be relevant to better understand the serotonin-dependent bidirectional control of LHb synaptic transmission.

Unlike excitatory transmission, much less is known about the serotonin-dependent modulation of GABAergic transmission in the LHb. Serotonin application decreased optogenetically-driven EPN-to-LHb IPSCs, and IPSCs evoked by extracellular stimulation (Hwang and Chung, 2014; Shabel et al., 2014). On the other hand, a chronic treatment with serotonin transporter inhibitors (SSRI), which typically elevates extracellular serotonin concentration, led to increased GABA/AMPA ratios and presynaptic GABA markers at EPN-to-LHb synapses (Shabel et al., 2014). This discrepancy might be due to the differential effect and adaptations that may occur between the acute activation of the receptors vs. the long-term changes triggered by the chronic SSRI treatment. Furthermore, it remains unclear whether the SSRI-dependent synaptic plasticity results from the direct serotonin modulation within the LHb or rather from wider circuit adaptations. These results set the stage for further investigation on the mechanisms underlying the serotonin-dependent synapse-specific plasticity in the LHb. An in-depth understanding of serotonin-dependent changes in synaptic transmission would allow to decipher their behavioral relevance and to assess their contribution to motivated behaviors or pathologies where this encoding is disrupted.

## Serotonin Modifies Output Firing of LHb Neurons

What could be the functional repercussions of serotonin release on LHb activity? Bath application of serotonin in rat brain slices induced a marked depolarization in the majority of LHb neurons. This effect was independent from synaptic neurotransmitter release as it was insensitive to tetrodotoxin and synaptic receptor antagonists. These effects on the membrane potential occurred along with an increase in LHb spontaneous neuronal firing. Serotonin-dependent increase of activity was more pronounced in cells recorded in the lateral division of the LHb (Zuo et al., 2016). This is in line with the reported predominant DRN input onto the lateral portion of the LHb (Muzerelle et al., 2016). Serotonin actions on the membrane potential and on LHb neuronal firing was mediated by postsynaptically expressed 5-HT2/3 receptors (Zuo et al., 2016). The effect of serotonin was dependent on transient receptor potential channels (TRP) and Ca^2+^ signaling, however a clear link between the 5HT receptors and its intracellular signaling remains unknown (Zuo et al., 2016). Moreover, increasing endogenous serotonin concentrations by Sert blockade induced a depolarizing current and increased the firing rate of LHb neurons, mimicking the effect of exogenous serotonin (Zuo et al., 2016; Figure 1).

Single unit recordings in anesthetized rodents indicate that a functional connection exists between the DRN and the LHb (Andersen et al., 1983; Dong et al., 1992). Indeed, local activation of the 5-HT2C receptors within the LHb transiently increased LHb spontaneous firing rate and bursting activity (Han et al., 2015). On the other hand, 5-HT1B receptor activation decreased serotonin release in the LHb as measured by microdialysis methods (Adell et al., 2001). This supports the above-described evidence for a presynaptic 5-HT1B receptor expression, and suggests a presynaptic modulation of serotonin release. Whether specific DRN-driven serotonin release occurs in the LHb, and whether this modulates LHb neuronal function remains to be explored.

Altogether, this evidence indicates the importance of serotonin in the modulation of LHb neuronal activity. Considering that the major effect exerted by serotonin is an increase in neuronal firing, serotonin release may therefore trigger avoidance or pathological states such as depressive symptoms characterized by LHb hyperactivity (Lecca et al., 2014).

## Concluding Remarks

That the LHb and the serotonergic system are anatomically interconnected is known since decades. However, the functional implications of this connectivity, and the specific modulation of synaptic transmission by serotonin release remain under-studied. The combination of optogenetics with pharmacology and electrophysiology has recently revealed that serotonergic signaling influences the activity of LHb neurons, as well as synaptic neurotransmission. Despite this evidence, the function, subterritorial expression and behavioral relevance of discrete 5-HT receptors within the LHb remain vague. This is partly hampered by the limitation in mimicking of physiological serotonin release and its consequences on LHb function. Indeed, the data so far rely on receptor pharmacology in acute brain slices. Future research needs to tackle the role of 5-HT receptors by employing genetic strategies, as well as electrochemistry or for instance by pushing the bioengineering of 5-HT receptors that would be activated by synthetic drugs or light.

While we have discussed the anatomical connections underlying serotonergic signals within the LHb, it remains unknown when and how serotonin release occurs in the LHb. The use of approaches assessing neuronal activity *in vivo* may represent a strategy to circumvent this problem.

The activity of serotonin neurons regulates a wealth of brain functions including emotions, appetitive and aversive stimuli or aggression (Audero et al., 2013; Hayashi et al., 2015; Teissier et al., 2015). Furthermore, perturbation of serotonin neurons firing and thereby serotonin release contributes to pathological states such as neuropathic pain, anxiety, mood disorders and addiction (Müller et al., 2007; Blier and El Mansari, 2013; Sagheddu et al., 2015). It is plausible that serotonin-dependent modulation of LHb neurotransmission may contribute to serotonin-driven behaviors in physiological conditions and pathologies that are characterized by aberrant LHb activity (Li et al., 2011; Meye et al., 2015; Lecca et al., 2016). It would be important to understand the relevance of the reciprocal connection of LHb-Raphe-LHb, and the repercussions onto specific LHb downstream systems, such as the dopamine system, a key player in motivational processing. A major effort on these topics would allow a better understanding of the role of serotonin in encoding aversive and rewarding stimuli. It may furthermore help to decipher the maladaptive mechanisms taking place in neuropsychiatric disorders characterized by perturbation of LHb and serotonin system function.

## Author Contributions

MM, AT and KV together contributed to the conceptualization of the manuscript and wrote the manuscript.

## Conflict of Interest Statement

The authors declare that the research was conducted in the absence of any commercial or financial relationships that could be construed as a potential conflict of interest.
